# The Role of Glucocorticoid Receptors in Podocytes and Nephrotic Syndrome

**DOI:** 10.11131/2018/101323

**Published:** 2018-04-24

**Authors:** Xuan Zhao, Daw-Yang Hwang, Hung-Ying Kao

**Affiliations:** 1 Department of Biochemistry, School of Medicine, Case Western Reserve University, 10900 Euclid Avenue, Cleveland, Ohio 44106, USA; 2 Division of Nephrology, Kaohsiung Medical University Hospital, Kaohsiung Medical University, Kaohsiung, Taiwan

**Keywords:** Glucocorticoid receptor, Podocyte, Nephrotic syndrome, focal segmental glomerulosclerosis

## Abstract

Glucocorticoid receptor (GC), a founding member of the nuclear hormone receptor superfamily, is a glucocorticoid-activated transcription factor that regulates gene expression and controls the development and homeostasis of human podocytes. Synthetic glucocorticoids are the standard treatment regimens for proteinuria (protein in the urine) and nephrotic syndrome (NS) caused by kidney diseases. These include minimal change disease (MCD), focal segmental glomerulosclerosis (FSGS), membranous nephropathy (MN) and immunoglobulin A nephropathy (IgAN) or subsequent complications due to diabetes mellitus or HIV infection. However, unwanted side effects and steroid-resistance remain major issues for their long-term use. Furthermore, the mechanism by which glucocorticoids elicit their renoprotective activity in podocyte and glomeruli is poorly understood. Podocytes are highly differentiated epithelial cells that contribute to the integrity of kidney glomerular filtration barrier. Injury or loss of podocytes leads to proteinuria and nephrotic syndrome. Recent studies in multiple experimental models have begun to explore the mechanism of GC action in podocytes. This review will discuss progress in our understanding of the role of glucocorticoid receptor and glucocorticoids in podocyte physiology and their renoprotective activity in nephrotic syndrome.

## 1. GR Signaling

Glucocorticoid receptor (GR) is a founding member of the nuclear hormone receptor (NHRs) that control homeostasis, differentiation, proliferation and animal development. NHRs bind their cognate hormones and regulate the expression of a complex genetic network, in which their coordinated activity defines the physiological, hormonal responses. A key function of NRs is to mediate transcriptional regulation in response to hormones and other metabolic ligands through the recruitment of an array of positive and negative regulatory proteins, referred to as co-activators or co-repressors.

**Figure 1:**
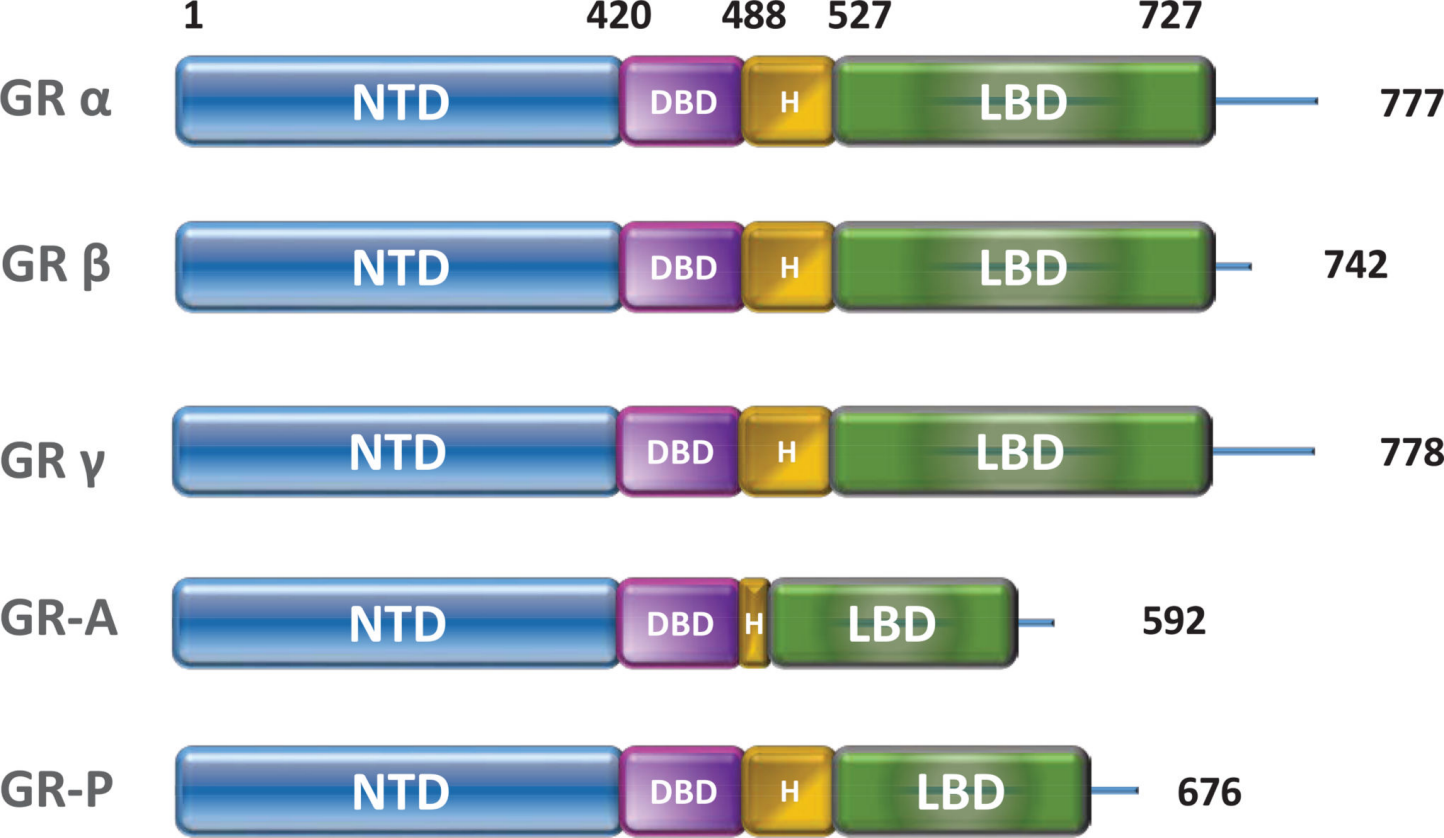
The GR family proteins Human GR harbors three functional domains: N-terminal domain (NTD), middle DNA-binding domain (DBD) and the C-terminal ligand-binding domain (LBD). The DBD and LBD are linked by the hinge region (HR). Alternative splicing of the *NR3C1* (gene encoding GR) gene generates the isoforms GRα, GRβ, GRγ, GR-A, and GR-P, which differ in size and sequence of HR and/or LBD.

GRα is composed of four functional domains, the N-terminal ligand-independent transactivation domain (NTD) or activation function 1 (AF-1), the DNA-binding domain (DBD), the flexible hinge region and the ligand-binding domain (LBD). The LBD contains 12 helices including the ligand-binding pocket (helices 3, 4, 5 and 12) and the AF2 domain (Figure 1). Glucocorticoid binding to the hydrophobic pocket of the LBD triggers a conformational change, thereby unmasking the LBD from the AF2 domain followed by subsequent co-activator binding [1, 2]. The AF1 and AF2 domains have been shown to activate transcription through its interaction with the basal transcriptional machinery and transcriptional co-activators [3].

**Figure 2:**
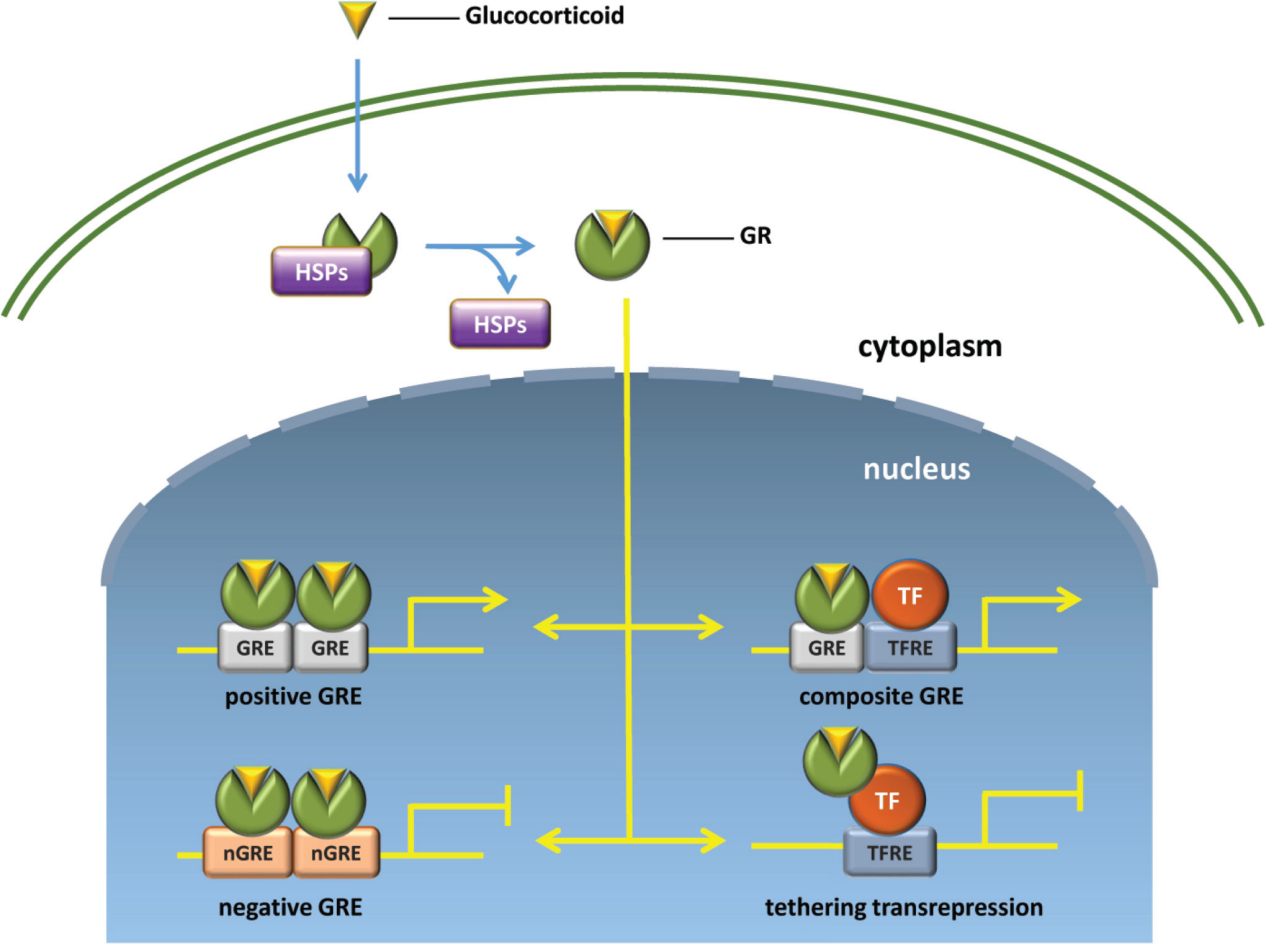
Molecular mechanism of GR signaling pathways Glucocorticoids diffuse across the cell membrane to the cytosol, where they bind GR. Glucocorticoid binding promotes dissociation of GR from chaperone proteins (HSPs) and subsequent nuclear translocation. Once in the nucleus, GR forms hetero- or homodimers and interacts with DNA to control gene transcription. Ligand-bound GR can lead to either activation or repression of gene transcription. TF: transcription factor; GRE, glucocorticoid response element; nGRE, negative glucocorticoid response element; TFRE: transcription factor response element.

Glucocorticoid signaling is primarily dependent on GR-mediated transcription and protein synthesis [4]. In the absence of hormone, the GR resides in the cytoplasm as part of a large multiprotein complex that includes chaperone proteins such as HSP90 [5, 6]. Upon ligand binding, GR dissociates from the chaperone proteins and translocates into the nucleus, where it regulates transcription through multiple distinct modes of action (Figure 2). As a homodimer, it binds a cognate DNA sequence present in enhancers containing glucocorticoid response elements (GREs) to activate gene expression [7, 8]. In addition to homodimerization, GR also directly interacts with MR or AR to form heterodimers [9]. Furthermore, ligand-bound monomeric GR binds composite GC-responsive regions with additional transcription factors such as signal transducer and activator of transcription (STAT), and cAMP response element-binding protein (CREB) and potently induce glucocorticoid-mediated gene expression [10–12]. The recruitment of several coactivators, including histone modifying enzymes and chromatin modulators promotes chromatin remodeling and subsequent transcription initiation [13–16]. The GR homodimers also bind specific sequences called negative GREs (nGREs) in the promoter region of several target genes and repress their transcription [17]. Lastly, in contrast to the dimer, ligand-bound GR monomeric is capable of transrepressing transcription through its interactions with other transcriptional regulators, such as nuclear factor kappa B (NF-κB) and activating protein-1 (AP-1). These interactions block co-activator recruitment and promote co-repressor recruitment, thereby altering chromatin structure and repressing target gene expression [18–20].

## 2. Glucocorticoids (GCs)

As a ligand-dependent transcription factor, the physiologic and pharmacologic action of GR is primarily mediated by the glucocorticoids (GC). The synthesis and release of GCs are under dynamic circadian regulation by the hypothalamic-pituitary-adrenal axis [21, 22].

Synthetic GCs are drugs that mimic the action of natural GCs. Dexamethasone (Dex), prednisone/prednisolone, and budesonide are the most commonly prescribed synthetic GCs [23, 24]. Synthetic GCs are prescribed for chronic inflammatory diseases, including autoimmune disorders, allergies, asthma and skin infections [25]. In addition to their anti-inflammatory properties, GCs have been used in conjunction with cancer chemotherapy to reduce side effects [26]. Importantly, synthetic GCs, such as Dex and prednisone/prednisolone, are therapeutically effective in treating nephrotic syndrome [27, 28]. Notably, it has been proposed that Dex can directly act on the glomerular podocytes contributing to its therapeutic effects [29].

## 3. GR Target Genes

Genome-wide analyses of GR-regulated genes and GR-binding sites in different cell types and tissues have recently been reported [30–32]. These experiments reveal the characteristics of genome-wide profiling of GR and genome-wide inventory of GR-binding sites. These results provide an exciting global view of the GR target genes and tissue-specific modes of GR action and potentially contribute to our understanding of glucocorticoid action. ChIP-seq studies showed that GR binding sites are not present in isolation but are often surrounded by binding motifs for other transcriptional factors such as AP-1 [33].

It is striking that GR selectively regulates transcription in a cell-specific manner. Chromatin accessibility is a significant contributor to the determination of the tissue-specific GR binding profiles and the primary determinant for tissue-specific chromatin accessibility is the cell type-specific expression of other transcription factors. Most GR target genes are involved in metabolism, signal transduction, inflammation and the immune response [34–36]. These GR target genes include both induced and repressed genes that are associated with known GC functions. Consistent with their ability to modulate the expression of inflammation-associated genes, GCs are widely used in medical therapy for immunosuppression and anti-inflammatory agents. However, GCs’ broad effects on different tissues can cause unwanted side effects such as bone loss and glucose dysregulation. It is hopeful that the information extracted from ChIP-seq and RNA-seq data in different tissues will provide mechanistic insights into a better understanding of GCs’ global effects and ultimately help develop agents that alleviate unwanted side effects.

## 4. Podocytes and Nephrotic Syndrome

### 4.1. Glomerular podocytes

**Figure 3:**
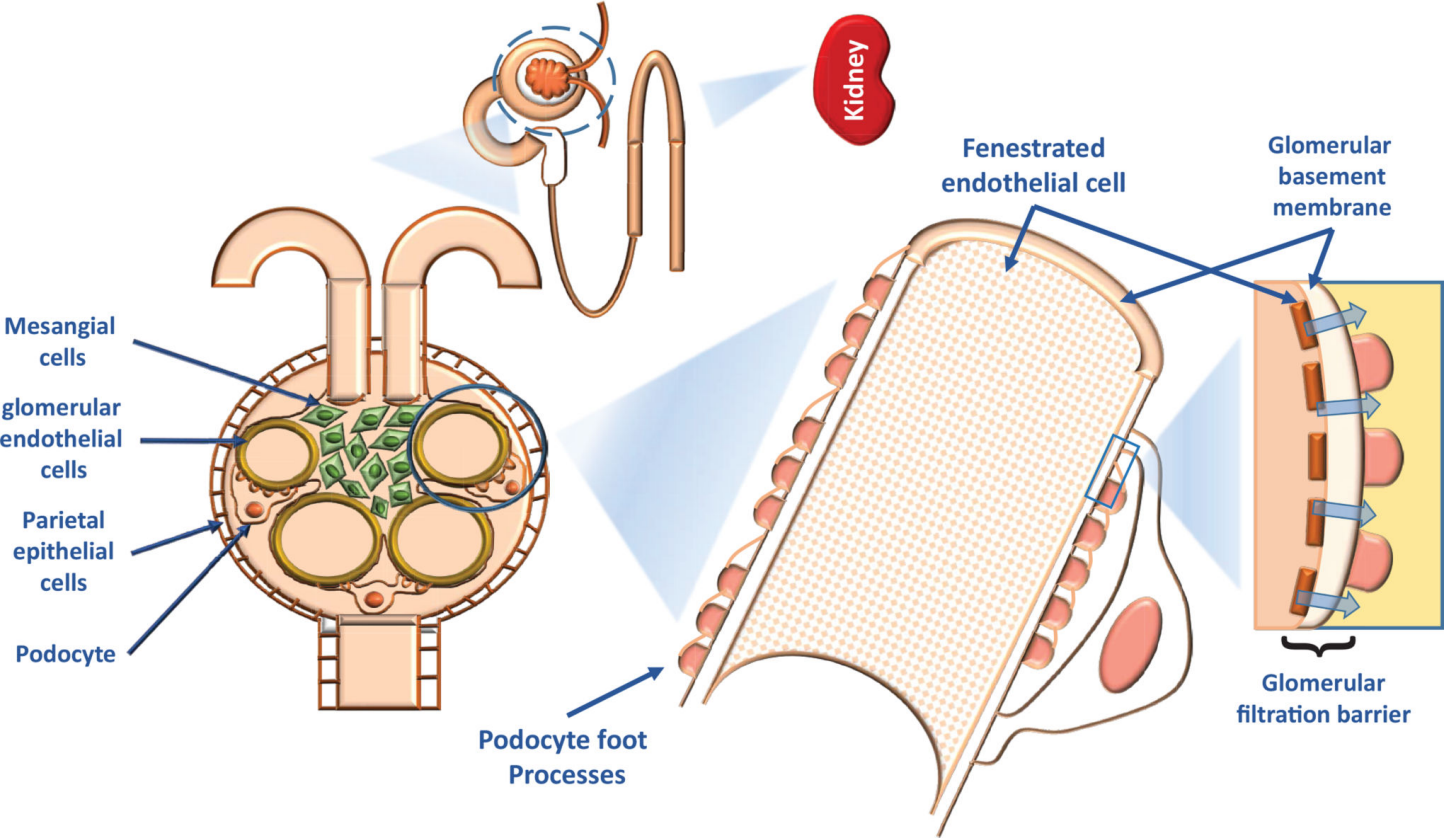
A diagram showing the structure and components of the renal glomerular filtration system, from the kidney to podocyte The glomerular filtration barrier consists of fenestrated endothelial cells, glomerular base membrane, and podocytes.

One of the crucial functions of the kidney is to remove toxins and metabolic waste while preventing proteins larger than albumin from entering the urine. The glomerulus is the functional unit required for blood plasma filtration and primary urine production [37]. Four distinct cell types assemble to form the glomerulus: glomerular endothelial cells, mesangial cells, podocytes, and parietal epithelial cells (PECs) (Figure 3, [38]). Podocytes are fully differentiated epithelial cells covering the outer surface of the glomerular basement member (GBM) and are critical for maintaining the integrity of the glomerular filtration barrier (GFB) [39]. The podocyte has a unique cell architecture that consists of an arborized cell body, primary processes, and secondary foot processes [40]. The long-interdigitated foot processes wrap around glomerular capillaries between adjacent podocytes and form the filtration slits, which are spanned by the slit diaphragms (SD), a highly specialized membrane-like cell-cell junctions. The cell body contains a nucleus and most of the cytoplasm, while the foot processes include primarily a dense network of actin filaments connected with an array of transmembrane proteins that link the SD and the GBM anchor proteins [41, 42]. The unique structure of the cell primary and secondary processes are maintained by the highly-organized cytoskeleton (Figure 3).

**Figure 4:**
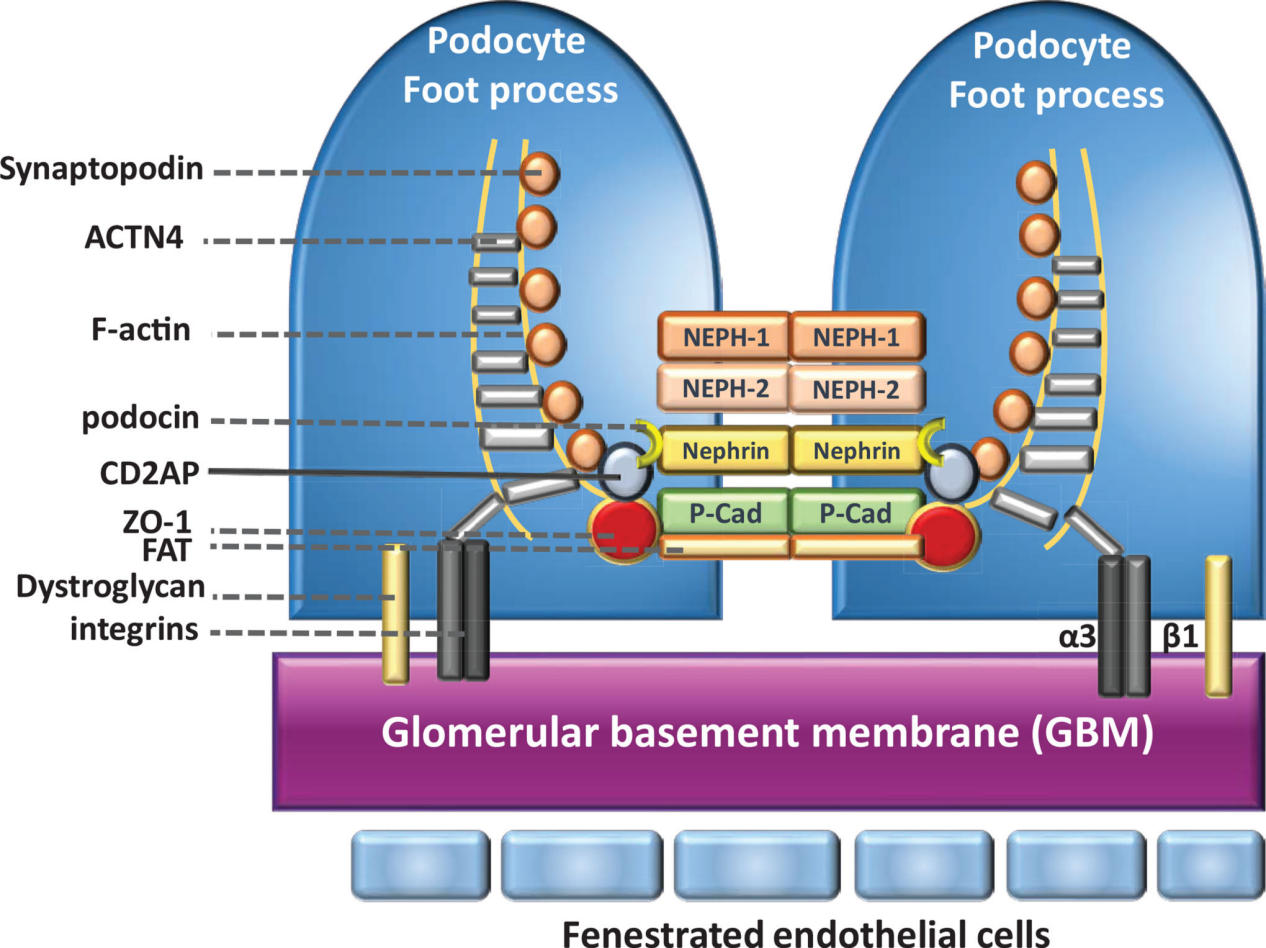
A schematic diagram depicting components of the podocyte slit diaphragm and foot processes and slit diaphragm proteins Proteins that make up the SD between adjacent foot processes are depicted. Nephrin, NEPH1, NEPH2, P-cadherin, and FAT are membrane-spanning proteins that have large extracellular domains that are important for signaling events that determine the structural integrity of podocyte foot processes. These proteins include the slit diaphragm interact with intracellular adapter proteins, including CD2-AP, ZO-1, Synaptopodin, and ACTN4. The adapter proteins bind to filamentous actin (F-actin). The adhesion molecules dystroglycan and α3β1 integrin anchor the podocyte to the underlying glomerular basement membrane (GBM).

The highly-specialized podocytes SD structure is in charge of macromolecular filtering and connects the podocyte actin cytoskeleton to transmembrane proteins and receptors and regulates plasticity of the foot process. As such, SD is a unique structure mediating cell-cell interactions, and possibly relaying extracellular signaling stimuli [42]. A growing number of molecular components of mature SD have been identified, many of which are components of tight and adherent junctions. Both nephrin and podocin are podocyte-specific proteins that are found only in the SD [43–45]. Other proteins associated with this unique structure include CD2-associated protein (CD2AP), transient receptor potential channel 6 (TRPC6), alpha-actinin 4 (ACTN4), P-cadherin, FAT1, synaptopodin (Synpo), α- and β-catenin, zonula occludens-1 (ZO-1), nephrin homologue NEPH-1, and Wilms’ tumour suppressor 1 (WT1) [46–53] (Figure 4). These podocyte proteins are associated with survival, differentiation, and unique cytoskeleton-dependent morphology of the podocytes [54, 55].

### 4.2. Podocyte injury

**Figure 5:**
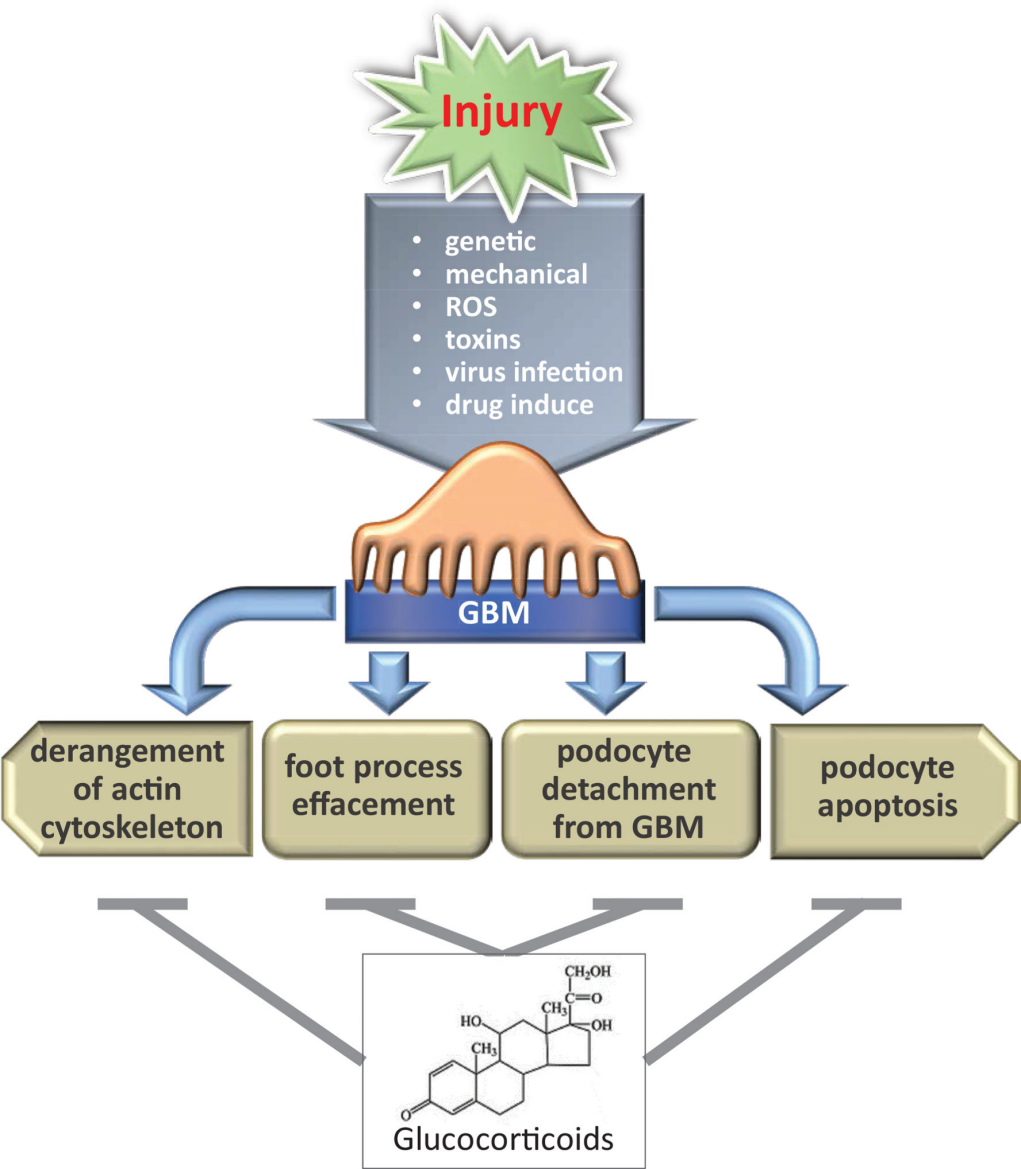
The mechanism of the podocyte injury and the protective effected by glucocorticoids Several causes are known to contribute to podocyte injury. After the injury, podocytes can undergo cytoskeleton derangement, effacement, detachment or apoptosis. The mechanisms by which glucocorticoids exerts its renoprotective effect involve several mechanisms that protect podocyte from injury.

Podocytes play a critical role in the preservation of the integrity of the GFB under normal conditions and are the target of many forms of physiological stress and pathological states. Podocytes respond to genetic, mechanical, reactive oxygen species (ROS), immunological stresses, toxins, viral infection and drugs [56, 57]. Podocyte injury occurs when excessive stress disrupts homeostasis. The beginning of podocyte injury includes derangement of the actin cytoskeleton [58, 59], loss of SD proteins and structural integrity, which lead to subsequent foot process effacement and podocyte detachment from GBM or apoptosis [60, 61]. It is widely accepted that podocyte injury results in proteinuria and nephrotic syndrome (Figures 3 and 5).

Upon injury, podocytes undergo apoptosis [62], which lead to a decrease in podocyte number. In the classical view, apoptosis has long been considered to be the primary cause of podocyte loss. Podocytes undergo apoptosis during the pathogenesis of the glomerular disease, as well as in mice exposed to PAN (puromycin aminonucleoside) treatment [62–64]. Podocyte detachment from the GBM is a terminal event in podocyte injuries, which can promote further glomerular damage [65–67]. The detachment of podocytes from GBM occurs in regions of sclerotic lesions of the glomerulus and consequently increases the appearance of podocytes and podocyte-associated molecules in urine.

### 4.3. Nephrotic syndrome

NS represents a term for a collection of conditions [68]. It is a kidney disorder that causes the body to excrete too much protein in the urine [69]. The key features of NS are proteinuria, hypoalbuminemia, hypercholesterolemia, and edema. In children, proteinuria is defined as more than 0.1g of urine protein per square meter of body-surface area per day (Note: proteinuria is age-dependent in the child, much higher in the neonate). In adults, the nephrotic syndrome is defined as a urine protein level of more than 3.5 g per day [70].

Based on kidney biopsies, NS patients can be diagnosed more specifically, including minimal change disease (MCD), focal segmental glomerulosclerosis (FSGS), membranous nephropathy and immunoglobulin A (IgA) nephropathy among others [71–74] (Table 1). FSGS can be further broadly classified as primary or adaptive. Primary FSGS is caused by monogenic alteration events, while adaptive FSGS, also referred to as secondary to FSGS, is associated with glomerular dysfunction associated with other diseases. This review will focus on primary FSGS. Normally, kidneys clear waste materials from the body and maintain a healthy balance of fluids and electrolytes in the blood. Upon the damages of the filtering units of the kidney, proteins that are usually kept in the plasma leak into the urine in large amounts. Various diseases, such as diabetes mellitus, hypertension, lupus erythematosus and viral infections, can damage glomeruli, resulting in proteinuria and NS [75–79]. Most NS in young children are idiopathic FSGS or frequently MCD, which is considered a less severe form of FSGS [80]. In adults, FSGS is the most common form of the glomerular disease [81] and a leading cause of the primary NS. FSGS accounts for 20% of NS in children and 40% in adults. FSGS is also the leading cause of glomerulonephritis-associated end-stage renal disease (ESRD) [82].

FSGS is viewed as a podocyte disease or “podocytopathy” [83, 84]. This is because mutations in several genes encoding components of the SD, cell-membrane, actin-cytoskeleton and signal transduction complexes in podocytes are associated with FSGS [85–91]. More than 50 genes have also been identified as the disease-causing genes for NS [92–100]. The goal of NS therapy is to preserve kidney function and achieve remission of proteinuria [101, 102]. GCs are more effective for the treatment of MCD, but commonly require adjunctive therapy with additional agents for FSGS patients (Table 1). The calcineurin inhibitors, such as cyclosporine and tacrolimus, are widely used in the treatment of steroid-resistant NS (SRNS), of which the majority are FSGS [103, 104]. The significant effects of calcineurin inhibitors are stabilization of the podocyte actin cytoskeleton, and subsequent reduction in proteinuria, independent of its impact on the immune system.

Increasing evidence from patients and experimental models have implicated an essential role for the immune system in the pathogenesis of idiopathic nephrotic syndrome. Several excellent reviews have thoroughly discussed this topic [105, 106]. Indeed, the chimeric anti-CD20 monoclonal antibody, rituximab, originally used to treat many B cell lymphomas, has beneficial effects in ameliorating proteinuria [107]. Taken together, the use of immunosuppressive therapy in the treatment of non-genetic forms of NS suggests a role for the impaired immunity in the pathogenesis of NS.

The NF-κB transcription factor family of proteins plays a crucial role in the regulation of the induction and resolution of inflammation. Accumulated evidence suggests the involvement of NF-κB activation induced by pathogenic agents in experimental NS models and NS patients. NF-κB activation has been demonstrated in glomerular cells such as podocytes, mesangial cells, tubular and endothelial cells upon renal injury or after exposure to inflammatory stimuli both *in vivo* and *in vitro* [108–110]. Several NF-κB-inducible genes and their encoded proteins including angiotensin II and cytokines, such as IL-1, IL-8, E-selection and MCP-1 are associated with the progression of glomerulonephritis, tissue injury in nephrotoxicity and other renal diseases, including glycosylated IgA [111–119]. Dysregulation of the activity of canonical NF-κB, p50/p65 (RelA), in podocytes has pathogenic consequences in glomerular diseases [120]. For example, activation of NF-κB contributes to HIV-associated nephropathy (HIVAN) [121]. This aberrant NF-κB activation specifically has a role in enhancing the effects of the TNF family of receptors on podocytes including the activities of Fas/FasL and TNFR2 [122]. Other reports indicate that activation of the ERK pathway and subsequent nuclear translocation of NF-κB are necessary for Ang II-induced TRPC6 accumulation and podocyte apoptosis [123] and that NF-κB activity mediates PAN-induced glomerular injury and proteinuria [124]. Collectively, these observations indicate that NF-κB is an important mediator of pathogenic processes in glomerulopathies and that balanced NF-κB activity is critical to maintaining glomerular integrity and function. Because NF-κB family proteins are present in most renal and the immune cells, the ability of GCs to transrepress NF-κB transcriptional activity in these cell types contributes to their overall efficacy when treating NS [125, 126].

## 5. The Effects of Glucocorticoid Therapy on Nephrotic Syndrome

Glucocorticoids have an essential role in podocyte development and treatments for nephrotic syndrome [127]. The physiologic and pharmacologic actions of GCs are mediated by GRα [128–130]. Ligand-bound GR induces or represses the transcription of target genes through direct binding to DNA or association with other transcription factors. Glucocorticoids have been used as immunosuppressive drugs for many diseases by reducing inflammation [131]. It has been a long-established clinical practice to use GCs to treat kidney disease. Recent studies in multiple experimental models have begun to explore the direct and indirect effects of GCs in podocytes to better understand its renoprotective activity as well as its unwanted effects.

The remission rates of GC therapy of NS vary between patients, depending on age, initial renal function, and the pathological features of NS [70, 132]. Based on their steroid responsiveness, patients are classified as steroid-sensitive and steroid-resistant. Genetic mutations that affect glomerular podocyte function, such as *NPHS1, NPHS2*, and *WT1* [133–137], account for most steroid-resistant cases and patients with genetic forms of steroid-resistance are less responsive to immunotherapeutic drugs. The circulating factor, soluble urokinase receptor, has been considered a cause for the development of SRNS [138, 139]. SRNS in adults has been defined as the persistence of symptoms after a 4-month trial of therapy and will inevitably progress to ESRD [140]. Alternative therapeutic strategies, including calcineurin inhibitor therapy, alkylating agents, and angiotensin-converting enzyme inhibitors, have been used to reduce proteinuria in steroid-resistant patients with FSGS [141].

Corticosteroid therapy has been used in childhood NS since the 1950s. GC therapy is more effective alone for children with MCD, but usually requires a combination with additional agents for adult NS [102, 142, 143] (Table 1). Children with NS are treated with oral prednisone for 2 to 3 months [81]. A combination of higher doses and increased duration of prednisone therapy can lead to enduring remission. Eighty percent of children with MCD respond to steroid therapy [143]. Thus, therapeutic decisions in children with SRNS are based on the underlying etiology [80, 144]. In contrast, adults with the NS usually undergo renal biopsy prior to the initiation of therapy. The renal biopsy is essential to determine the nature and severity of the glomerular processes and to clarify the type and causes of the glomerular nephropathy [145]. Patients whose biopsies demonstrate more cellular lesions are associated with a poor therapeutic response [146, 147]. Approximately 35% percent of adult patients fail to respond to initial steroid treatment and do not attain remission [102, 142]. A standard procedure for adults with FSGS is high dose glucocorticoid therapy for a significantly longer duration [148]. For patients who have a well-preserved renal function, initial high-dose prednisone is given for 3 to 4 months. However, complete remission rates for glucocorticoid therapy in adults with primary FSGS was quite disappointing [142, 148]. Consequently, there is less evidence to support steroid therapy for adaptive or genetic forms of adult FSGS patients. Thus, understanding the mechanism underlying steroid-resistance is an urgent matter for NS therapy.

### 5.1. The mechanisms underlying steroid-resistant nephrotic syndrome (SRNS)

Our current understanding of the mechanism underlying SRNS remains rather limited. This is in part due to the broad effects GCs have on multiple cell types through distinct mechanisms. Furthermore, the complexity and heterogeneity of SRNS make it difficult to establish correlations with genetic alterations. It is estimated that over 50 genes are associated with SRNS showing a different spectrum of phenotypes ranging from autosomal recessive to dominant and their onset from within 3 months after birth to late in adulthood [149]. Mutations in several early onset SRNS genes encode podocyte slit-diaphragm-associated proteins, indicating an important role for podocytes integrity in the pathogenesis of SRNS. Notably, several of these genes including NPHS1 [130], NPHS2 [150], TRPC6 [151] and CD2AP [152] are induced by Dex. However, Dex may have unwanted effects by inducing the expression of genes that promote further injury to glomeruli [153, 154]. Furthermore, mutations of *ACTN4*, which encodes a well-known cytoskeletal protein, are tightly associated with steroid-resistant FSGS. Our lab has recently reported that ACTN4 is a transcriptional coactivator for GR and FSGS-associated mutations are defective in GR-mediated transcriptional regulation [153]. Moreover, earlier reports indicated *ACTN4* deficiency is found in multiple human primary glomerulopathies including sporadic FSGS, MCD, and IgA nephropathy [155–158]. It will be intriguing to learn whether other SRNS-associated genes play a physiological role in GR signaling networks or are GR downstream targets.

## 6. The Role of Glucocorticoids in Podocytes

### 6.1. The direct effects of GCs on podocytes

The glucocorticoid receptor, as well as the major GR transcriptional cofactors, are expressed in human podocytes [18, 127, 130]. In order to determine whether podocytes are the key cell type that responds to glucocorticoid therapy, recent studies in murine and human podocytes have shown that Dex directly regulates podocyte morphology and function (Figure 5). Mathieson et al. first evaluated the direct effect of Dex on immortalized human podocytes (HPCs) *in vitro*. Dex treatment (100 nM ~ 10 µM) up-regulated *NPHS1* expression, and down-regulated *VEGF*, as well as *CDKN1A* (cyclin kinase inhibitor p21) and inflammation-associated cytokines, such as *IL6*. A proteomic analysis also identified proteins with known roles in protecting podocytes from injury and found them to be up-regulated by Dex in cultured murine podocytes [154]. These up-regulated proteins include proteins involved in the orchestration of the actin cytoskeleton and stress responses. Using microarray analyses, our lab showed that Dex induces *SERPINE1* (encoding Plasminogen Activator Inhibitor Type 1 or PAI-1) and *CCL20* mRNAs [153]. PAI-1 is present in trace amounts in healthy kidneys but increases in a wide variety of both acute and chronic diseased kidneys. Reduced PAI-1 activity has been shown to be protective of albuminuria and glomerulosclerosis in experimental diabetes [159], while *CCL20* is upregulated in patients with progressive IgA nephropathy [160]. Thus, Dex potentially exhibits unwanted effects by inducing genes including *SERPINE1* and *CCL20*, which may cause damage to podocytes or glomeruli. Our studies also uncovered that GR crosstalks with a broad range of signaling pathways, primarily the NF-κB, STAT and TGFβ, but also the inflammatory response, cell migration, and angiogenesis [161]. These data are consistent with the mechanism underlying transactivation and transrepression by GCs (Figure 2). GCs are considered to have immunosuppressive and anti-inflammatory effects. It exerts the anti-proteinuria effect not only by suppressing but also through protecting podocyte integrity. Recently, RNA-seq analysis revealed that Dex-regulated genes are linked to cytoskeleton-related processes, podocyte differentiation, pro-inflammatory cytokines and growth factors [162]. Collectively, these results advance our knowledge of the molecular mechanisms by which GCs exert their therapeutic effects on podocytes and potential targets for unwanted effects.

### 6.2. GCs and podocyte injury

GCs have significant effects on podocytes. Podocytes are therefore an important therapeutic target for the treatment of NS caused by genetic mutations or environmental stress. Current evidence suggests that GCs protect podocytes from experimental injuries induced by PAN, Adriamycin (ADR), or protein-overload [163, 164]. In an experimental podocyte injury model, up-regulation of *TRPC6* was shown to contribute to Angiotensin II (Ang II)-induced podocyte injury [165]. Notably, Dex treatment significantly reduced PAN-induced *TRPC6* expression in rat and cultured murine podocytes [166]. Furthermore, Agrawal et al. showed that GCs reduced PAN-induced proteinuria in rats, in part, by elevating the expression of glomerular synaptopodin and nephrin, and reduced COX-2 expression in rats [167]. Serum albumin overload in rats has also been reported to not only induce structural and pathological changes in podocytes [168, 169], but also increase pro-inflammatory genes *COX-2, MCP-1, CXCL1*, and the stress protein HSP25 expression in both rat glomeruli and cultured podocytes [170]. Similarly, GCs inhibit serum albumin-induced *COX-2* expression via its transrepression activity on NF-κB. GCs are also implicated in activating glomerular antioxidant enzymes and protecting glomeruli from reactive oxygen species (ROS)-mediated injuries in PAN-induced nephrosis in rats [171]. Using zebrafish and cultured HPCs, a recent study demonstrated that GCs ameliorate PAN-induced podocyte injury by down-regulating caveolin-1 expression and overexpression of caveolin-1 impaired normal podocyte function [172]. In summary, podocyte injury can be relieved by GC treatment in animal models and cultured human podocytes, in part, through the ability of GCs to regulate its target gene expression.

### 6.3. GCs and actin-filament stabilization

As mentioned earlier, the podocyte actin cytoskeleton is a key component of the complex architecture of the slit diaphragm [37, 40]. GCs protect and enhance recovery of cultured murine podocytes through its ability to stabilize actin filaments [128]. Dex treatment induces a significant increase in the activity of the actin-regulating GTPase RhoA and thereby increases total cellular polymerized actin, stabilizing actin filaments, and blocking PAN-induced disruption of actin filaments [128, 173–175]. Additionally, a recent study in cultured podocytes indicated that Dex could protect podocytes from ADR-induced actin rearrangements [163]. These reports imply that the beneficial effects of GCs in treating renal disease, at least in part, results from enhancing the stability of podocyte actin filament.

### 6.4. GCs and podocyte apoptosis

One of the beneficial features of GC action is the prevention of podocyte apoptosis [164]. GCs inhibit apoptosis by restoring *Bcl-2* expression, reducing p53 levels and inhibiting nuclear translocation of apoptosis-inducing factor (AIF) in PAN-treated cultured podocytes [164]. These activities are mediated, in part, by the blockade of PAN-mediated reduction of extracellular signal-regulated kinase (ERK) phosphorylation in response to Dex treatment [176]. PAN also inhibits PI3K/Akt signaling, and Dex treatment restores the PI3K/Akt signaling, which promotes the activity of anti-apoptotic proteins [152]. In another study, prednisone treatment was shown to reduce podocyte apoptosis. Dex also increased podocyte progenitors by activating ERK signaling in an FSGS mouse model induced by a cytotoxic anti-podocyte antibody [177]. Thus, GCs not only inhibit podocyte apoptosis but also increase the number of podocyte progenitors to prevent podocyte loss.

### 6.5. Animal knockout models

Renal epithelial cells include podocytes, parietal epithelial cells (PECs), and tubular cells. Using Pax8-*Cre/GR*^f l/f l^ mice, Kuppe et al. generated kidney epithelial cells-specific *Nr3c1* (GR) knockout mice [173]. These animals show no apparent abnormality in kidney development, indicating that renal epithelial GR is dispensable for kidney development. In a nephrotoxic serum (NTS)-induced glomerulonephritis (GN) mouse model, podocytes are injured and PECs become strongly activated. High-doses of GCs significantly improved NTS-induced renal dysfunction. Remarkably, Pax8-*Cre/GR*^f l/f l^ mice are resistant to NTS-induced GN, showing no or little albuminuria or cellular crescent formation. This observation is accompanied by fewer activated PECs, suggesting that GR promotes NTS-induced activation of PECs. This beneficial effect is also observed in NTS-treated mice exposed to mifepristone, a partial GR antagonist. Taken together, these data demonstrate a role of GR in the pathogenesis of NTS-induced GN, possibly due to a role of GR in activating PECs.

Using a *podocin*-Cre transgene, Zhou et al. have established podocyte-specific GR knockout (pGRKO) mice [174]. Consistent with renal epithelial-specific pGRKO mice, these animals showed no developmental phenotype and did not develop proteinuria under physiological condition. However, upon a challenge with by lipopolysaccharides (LPS) or NTS, pGRKO mice demonstrated severe proteinuria compared to control littermates. These observations support a critical role of podocytes GR in the maintenance of kidney function in response to LPS- and NTS-induced glomerular injury.

The recent literature further demonstrates that podocyte Krüppel-Like Factor 15 (Klf15) [178] and serum- and glucocorticoid-inducible kinase 3 (SGK3) [179], both of which are Dex-inducible genes, play essential roles in GC-mediated beneficial effects in response to LPS- or PAN-induced podocyte injury. In summary, these studies demonstrate an essential role for podocyte GR in response to injury as well as in the therapeutic effects of GCs.

## 7. Conclusion

It has been a longstanding accepted protocol to use GCs to treat NS. GCs have beneficial effects on patients with NS due to their ability to stabilize actin-filaments and to protect podocytes from apoptosis. Nonetheless, steroid-resistance and unwanted side effects associated with GC treatments are unacceptable and are an issue that needs to be addressed. The fundamentals surrounding this central issue include: 1) the targets of the GCs in podocytes, 2) the complexity of the molecular mechanisms underlying pathogenesis of NS and how they respond to GCs differently, 3) the benefits of combination therapy and 4) the molecular mechanisms by which GCs regulate physiology of different cell types in the glomerulus.

With pGRKO mice and the newly developed NUTRAP (Nuclear tagging and Translating Ribosome Affinity Purification) mouse strain [180], identification of GR-regulated gene networks in podocytes has become possible. A better understanding of the function of podocyte GR target genes will undoubtedly provide insight into the pathogenesis and treatment of NS.

The Nephrotic Syndrome Study Network (NEPTUNE) is a collaborative consortium that aims to develop a translational research framework for NS. This database contains multiple molecular and clinical data sets associated with samples collected from adults and children with NS that include MCD, FSGS, and membranous nephropathy [181]. This provides an unmatched resource to understand the mechanisms and pathways involved in NS. Integration of the data sets across the genome-phenome continuum, quantitative histology, rigorous clinical phenotypes and clinical outcomes will enable clinicians and researchers to better study genetic mutations associated with human kidney diseases [182]. Notably, this clinical information including steroid sensitivity will provide a wealth of critical data that will allow basic scientists to formulate and test hypotheses and ultimately help develop effective treatments for NS patients.

## Figures and Tables

**Table 1:** The pathology and steroid responses for nephrotic syndrome MCD, minimal change disease; FSGS, only include primary (idiopathic); MN, (primary) membranous nephropathy; IgAN, IgA nephropathy; IST, immunosuppressive therapy; Effective, means decreased proteinuria and/or slowing the progression of renal function.

	Pathology	Steroid response
MCD	foot processes effacement	Very good
FSGS	foot processes effacement and perihilar or sclerosis	Effective but may need other IST; relapse and resistant occurs
MN	Subepithelial deposition of the basement membrane on the outer surface of the capillary wall.	Effective, in combination with other IST
IgAN	IgA immune complex deposition in the mesangium	Effective, but with significant adverse effects
